# Haptic adaptation to slant: No transfer between exploration modes

**DOI:** 10.1038/srep34412

**Published:** 2016-10-04

**Authors:** Loes C. J. van Dam, Myrthe A. Plaisier, Catharina Glowania, Marc O. Ernst

**Affiliations:** 1 Cognitive Neuroscience / Cognitive Interaction Technology Center of Excellence (CITEC), Bielefeld University, Universitätsstraße 25, 33615 Bielefeld, Germany; 2 University of Essex, Wivenhoe Park, Colchester CO4 3SQ, UK; 3 Department of Human Movement Sciences, Research Institute MOVE, Vrije Universiteit Amsterdam, Van der Boechorststraat 9, 1081 BT Amsterdam, the Netherlands; 4 Applied Cognitive Psychology, Ulm University, Albert-Einstein-Allee 47, 89081 Ulm, Germany

## Abstract

Human touch is an inherently active sense: to estimate an object’s shape humans often move their hand across its surface. This way the object is sampled both in a serial (sampling different parts of the object across time) and parallel fashion (sampling using different parts of the hand simultaneously). Both the serial (moving a single finger) and parallel (static contact with the entire hand) exploration modes provide reliable and similar global shape information, suggesting the possibility that this information is shared early in the sensory cortex. In contrast, we here show the opposite. Using an adaptation-and-transfer paradigm, a change in haptic perception was induced by slant-adaptation using either the serial or parallel exploration mode. A unified shape-based coding would predict that this would equally affect perception using other exploration modes. However, we found that adaptation-induced perceptual changes did not transfer between exploration modes. Instead, serial and parallel exploration components adapted simultaneously, but to different kinaesthetic aspects of exploration behaviour rather than object-shape per se. These results indicate that a potential combination of information from different exploration modes can only occur at down-stream cortical processing stages, at which adaptation is no longer effective.

A hallmark of haptic (touch) perception is that it inherently requires active exploration of the objects around us. To do so however, a variation of different exploration behaviours can be employed. For example, for estimating the slant of a surface, one may place the entire hand or multiple fingers on the surface in which case the static posture provides a cue to slant. Since information from multiple locations on the hand is available simultaneously, we call this Parallel Exploration. Alternatively, surface slant can be sampled by moving a single finger along the surface. In this case the finger’s position information is integrated over time. Therefore, we call this Serial Exploration.

While different exploration modes can provide complementary information about different haptic object properties (e.g. for judging temperature it is best to use static contact and for estimating local surface texture contour following or rubbing works best)^1^, our perception of global object shape appears to be largely invariant under the serial or parallel exploration modes. This is true at least when sampling occurs globally (i.e. along a large portion of the object) and not only locally^1^,^2^. Moreover, the Parallel and Serial Exploration modes have been shown to assess the same low-level object features, such as local surface slant, for determining object shape^3^. Additionally, global object shape information received through the Parallel and Serial Exploration modes should be highly correlated in our daily experience, since we often explore the shape of objects by moving our whole hand across them: this way a combination of serial and parallel exploration is employed. Does this mean that the information obtained through parallel and serial exploration is also automatically combined into a shared representation of shape in the brain?

The merged processing of parallel and serial information would be in line with findings that show that proprioceptive information is combined with cutaneous touch information early in the somatosensory cortex^4^. Moreover, such a unified representation for low-level perceptual features, such as size, slant, and motion is a general hallmark of (multi)sensory perception (e.g. refs 5, 6, 7, 8). This suggests that the neural pathway for processing low-level perceptual features is shared across the senses and recent neurophysiological evidence suggests that such sensory combination occurs in the sensory cortex^9^.

To investigate the extent to which haptic parallel and serial exploration information may share such a common neural pathway for slant perception, we used a perceptual adaptation-and-transfer paradigm (see e.g. ref. 10 for a review). Adaptation occurs when exposed to the same sensory input, here surface slant, for a prolonged period of time. As a result, neurons in the periphery and the cortex that code for such input change their response gain (e.g. ref. 11). This in turn changes the perception of a subsequently presented test-stimulus that activates the same neural populations (adaptation aftereffect). Thus, perceptual adaptation can be used to make inferences about the existence of neural processes, and for this reason is often referred to as the “psychophysicist electrode”^12^.

In the present study participants adapted to surface slant either by moving one finger over the surface (Serial Adaptation) or by statically placing two fingers on the surface (Parallel Adaptation). In both cases this should lead to a change in the angle at which a surface feels level (adaptation aftereffect). Moreover, if serial and parallel exploration share the same low-level feature-based neural representation, the adaptive state should be independent of the exploration mode used for adaptation. This means that after adaptation using one exploration mode, the angle at which a surface feels level should also have changed when the other exploration mode is used for testing (adaptation transfer). Such adaptation aftereffects have previously been shown to transfer between vision and touch^7^ and between the two hands^13^.

Surprisingly however, whereas our results confirmed clear adaptation aftereffects within each exploration mode, we found no transfer between the Parallel and Serial Exploration modes. Instead, serial and parallel exploration could be adapted independently at the same time. This suggests that these exploration modes are processed in largely segregated neural pathways throughout the early stages of sensory processing.

## Materials and Methods

### Setup

**Figure 1:**
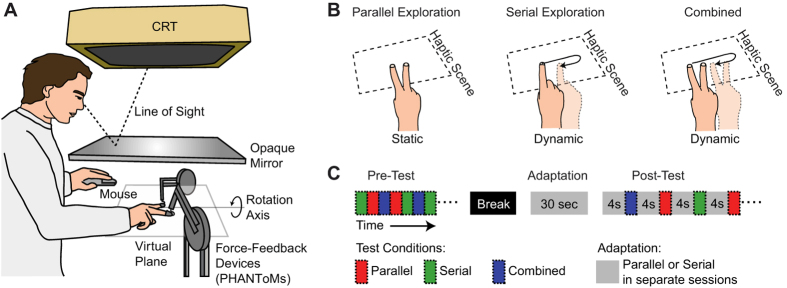
Setup and experimental procedure. (**A**) Experimental setup. Haptic surfaces were rendered using two PHANToM force-feedback devices. Participants responded which side felt higher, left or right, by pressing one of two buttons on a standard computer mouse. The experimental condition for the upcoming trial was presented by a colour-code message on a CRT monitor, which was viewed via a mirror. Direct vision of the hand or the PHANToMs was prevented by a black drape covering the haptic workspace underneath the mirror. (**B**) Experimental conditions. For Parallel Exploration participants placed two fingers on the surface and kept static contact. For Serial Exploration they moved a single finger over the surface. For Combined Exploration (i.e. serial and parallel) participants placed two fingers on the surface and moved them laterally. (**C**) Experimental procedure. In the pre-test the Parallel, Serial and Combined Testing trials were intermixed. After a short break the session continued with 30 sec of main adaptation to ±10 deg. surface slant. During the post-test the three exploration trial types were again intermixed, but each trial was preceded by 4 sec top-up adaptation. There was one session each for Parallel and Serial Adaptation.

Participants were seated behind a haptic workbench consisting of two PHANToM force-feedback devices (PHANToM premium 1.5, SensAble Technologies, Inc. Woburn, MA), one to each side of the experimental setup (Fig. 1A). A chin rest ensured the alignment between the participant's body midline and the centre of the haptic space. Participants placed the index and middle fingers of their preferred hand into thimble-like holders attached to each PHANToM. In this way, forces could be generated independently for each finger to render the surfaces and the positions of the fingers could be recorded. A CRT monitor (Sony CPD G500/G500J, Sony Europe Limited, Weybridge, UK; 140 Hz) was used to display information concerning the exploration mode to be used on the upcoming trial. A standard two-button computer mouse was used as a response device.

### Stimuli and Procedure

Stimuli were haptically rendered surfaces, using the PHANToM force-feedback devices. For test trials exploration time was always 1 sec and there were three separate exploration conditions: Serial, Parallel and Combined Exploration. For Serial Testing participants (freely) moved their index finger left-right across the surface to feel its slant (Fig. 1B, middle). The extent to which participants could move the index finger was limited to 7 cm to the left of the body midline and 7 cm to the right of the body midline. In the Serial Test condition, the PHANToM attached to the middle finger was switched off such that no surface information was available to the middle finger.

For Parallel Testing the surface was rendered via force feedback to both the index and the middle fingers, and participants kept static contact with the surface (Fig. 1B, left). To investigate how serial and parallel information may interact when both are available, we included the third Combined Exploration mode as a test condition (Fig. 1B, right). For Combined Testing participants received force feedback to both index and middle fingers (parallel information) but additionally moved both their fingers laterally across the surface (serial information). The movements in this case were again limited to 14 cm around the body midline.

Before each trial, participants were notified by a colour code covering the entire screen which exploration mode to use for the upcoming trial: Serial (green), Parallel (red), or Combined (blue). To start a trial, participants lifted their fingers to at least 35 mm above the area where the virtual surface would be rendered. At this time the colour instruction cue disappeared. Next participants self-initiated the 1 sec exploration period by lowering their fingers towards the surface to explore it. The exploration period started as soon as the participant first touched the virtual surface. When the exploration period had elapsed the haptic rendering of the surface was switched off. The participants’ task was to judge which side of the slanted surface felt higher, left or right (corresponding to the surface rotation axis shown in Fig. 1A). A surface perceptually at level will provide 50% left and 50% right responses, which is referred to as the point of subjective equality (PSE). Participants entered their response by button press on a standard computer mouse with their non-dominant hand after which the colour cue indicating the exploration mode for the next trial was shown.

We determined the PSE using an adaptive 1-up/1-down staircase procedure. That is, within each staircase the slant presented on the current trial depended on the slant on the previous trial and the corresponding left or right side higher response. In case of a “left”-response on the previous trial the surface was slanted more rightward on the current trial and vice versa. This way the slant converged towards the PSE across trials. Initially the step size between trials in each staircase was 8 deg., after 2 reversals the step size decreased to 4 deg. and after another 2 reversals the step size was 2 deg. for the remainder of the staircase procedure. A staircase was terminated after 12 reversals in the responses.

To quantify the effect of slant adaptation we compared the change in PSE between pre- and post-adaptation test results. In both the pre- and post-test phases there were two staircases for each exploration mode. One staircase started at +20 deg., the other at −20 deg. to control for possible hysteresis effects within the staircase procedure. This meant that in total there were 6 staircases for each phase (3 exploration modes x 2 staircases for each mode). Trials for these 6 staircases were presented randomly intermixed.

Figure 1C shows a schematic timeline of the experimental procedure. After the pre-test participants took a 5 to 10 minute break. After the break, participants were first presented with the adaptation stimulus for 30 sec. The adaptation surface had a slant of ±10 deg., counterbalanced across participants (care was taken that this was done for left and right-handed participants separately in order to also counterbalance for symmetry with respect to the dominant hand). The colour cue indicated which exploration mode to use. During adaptation there was no additional perceptual task. Next the post-test started for which again 6 staircases (3 exploration modes x 2 staircases for each mode) were presented randomly interleaved. The only difference between pre- and post-test trials was that during the post-test each trial was preceded by a 4 sec top-up adaptation interval. Top-up adaptation periods were again preceded by a colour cue. After top-up adaptation a second colour cue indicated which exploration mode to use on the upcoming test-trial.

Participants completed two sessions, one each for the Serial Adaptation and the Parallel Adaptation conditions. The procedure was the same except for the exploration mode that was used (serial or parallel) during the adaptation periods. For each participant the direction of the 10 deg. slant (left or right) of the adapting stimulus was the same on both sessions in order to prevent within-participant directional effects from influencing the results. The order of the two sessions as well as the direction of slant was counterbalanced across participants. One session (including the break) took approximately 45 minutes to complete. The two sessions were performed on separate days.

### Analysis

**Figure 2:**
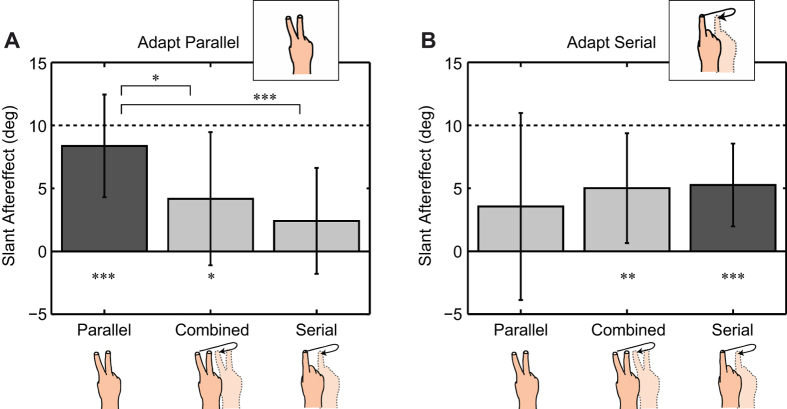
Experimental results. (**A**) Results for Parallel Adaptation and transfer (N = 14). (**B**) Results for Serial Adaptation and transfer (N = 14). Participants adapted to a surface slant of 10 deg. (dashed lines). Bars and error bars indicate the means and standard deviations across participants, respectively. Dark grey bars indicate the adapted condition, light grey bars the transfer conditions. Stars underneath the bars indicate significant adaptation after/transfer effects (comparison against zero) after Bonferroni-correction. Stars linking bars at the top indicate significant differences after Bonferroni correction between test conditions within a session.

To obtain the PSE, we fitted psychometric curves to the combined results of the 2 staircases for each exploration mode and test-phase. To fit the psychometric curves (cumulative Gaussians) we used the psignifit toolbox for Matlab^14^. The PSE was determined by taking the 50% cut-off of the obtained fits. We determined the size of the aftereffect by subtracting the pre-test PSE from the post-test PSE for each exploration mode. If the adaptation had no effect for a given exploration mode the difference between the pre- and post-tests should be zero. Therefore, to test for significant after/transfer effects for each individual exploration mode we tested the post/pre-test differences against zero using Bonferroni corrected t-tests (significance level = 0.017; stars below the bars in Fig. 2). Additionally, to test for significant differences *between* exploration modes we used repeated measures ANOVA's and posthoc Bonferroni-corrected paired t-tests (significance level = 0.017; stars linking the bars in Fig. 2). These tests were performed separately for each of the Serial and Parallel Adaptation sessions.

**Figure 3:**
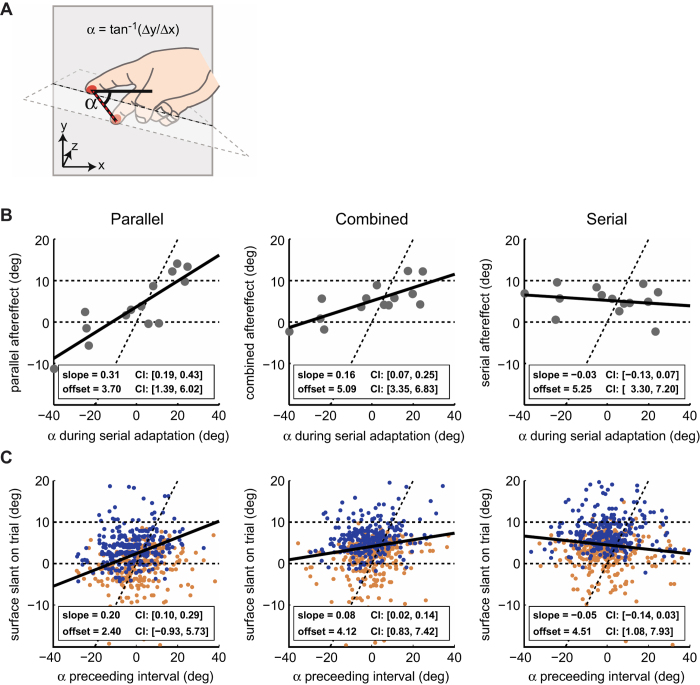
Serial Adaptation: Analysis of hand posture. (**A**) The posture angle α is obtained by comparing the axis between the positions of the index and middle fingers with level orientation. (**B**) Between participants analysis. The results for each of the three exploration modes (left: Parallel, middle: Combined, right: Serial) are plotted versus the average posture angle α during the Serial Adaptation phases for each individual participant. Grey dots represent the individual participants. The black line shows the linear regression. (**C**) Within participant trial-by-trial analysis. Dots represent single trials pooled across participants (for illustrative purposes the centres of the individual participants’ dot clouds were aligned for this graph). Blue dots indicate right-side higher responses, red dots left-side higher responses. The black line indicates the average Line-of-Subjective-Equality (LSE) across participants. Dashed lines in (**B**) and (**C**) represent predictions in terms of no aftereffect (horizontal line at 0 deg.), full aftereffect with respect to the surface slant during Serial Adaptation phases (horizontal line at 10 deg), and perception fully governed by the posture angle α (diagonal line) during adaptation. The results indicate a significant effect of hand posture during Serial Adaptation, on subsequent Parallel or Combined Testing (left and middle panels in **B**,**C**).

Besides the participants’ responses (left or right side of the surface felt higher), we were interested in the hand postures used during adaptation. Hand posture is the cue that provides slant information when using parallel exploration, but it was unrestricted during serial exploration. To obtain a measure for hand posture we recorded participants’ exploration movements using the position encoders of the PHANToM devices. The positions of the index and middle fingers were recorded every 21 ms. To quantify hand posture, we obtained the angle (α) between the orientation defined by the positions of the index and middle fingers relative to level (see Fig. 3A). We will refer to this as the “posture angle α” for the remainder of the manuscript.

### Participants

Sixteen participants performed both adaptation sessions of the main experiment (2 authors: LD and CG, and 14 students of Bielefeld University; 9 female; mean age 24; 2 left-handed). The study was conducted in accordance to the Declaration of Helsinki. Ethical approval was obtained from the University of Bielefeld ethics committee. Participants provided written informed consent and were compensated with 6€/hour of participation. The results of 2 right-handed participants were discarded since one or more of their staircases did not converge within 40 trials.

## Results

After adaptation the angle at which the surface felt level had changed significantly if the same exploration mode was used for both test and adaptation. This is evident for both Parallel Adaptation (Fig. 2A, dark grey bar, comparison to zero t(13) = 7.68; p = 0.000003; d = 2.05) and Serial Adaptation (Fig. 2B, dark grey bar, t(13) = 5.99; p = 0.000045; d = 1.60).

After Parallel Adaptation (Fig. 2A) the aftereffect for Parallel Testing was even complete. That is, the aftereffect did not differ from the adapted slant of 10 deg. (t(13) = −1.49; p = 0.16; d = 0.40). However, the results for both Serial Testing and Combined Testing after Parallel Adaptation were significantly smaller than the aftereffect for Parallel Testing (F(2, 26) = 11.3; p = 0.0003; η^2^ = 0.46; paired t-tests t(13) = −5.15; p = 0.00019; d = 1.38 and t(13) = −2.91; p = 0.012; d = 0.78, respectively). The result for Combined Testing was significantly different from zero, indicating that there was at least partial transfer (t(13) = 2.96; p = 0.011; d = 0.79). For Serial Testing there was no significant difference from zero after Parallel Adaptation (t(13) = 2.15; p = 0.051; d = 0.58), indicating no significant transfer from Parallel to Serial Exploration.

After Serial Adaptation (Fig. 2B) there was a significant aftereffect for Serial Testing (comparison to zero t(13) = 5.99; p = 0.000045; d = 1.60), however, Serial Adaptation was incomplete since the aftereffect differed significantly from the 10 deg. adaptation slant (t(13) = −5.39; p = 0.00012; d = 1.44). Serial Adaptation transferred to the Combined Testing condition (comparison to zero for Combined Testing: t(13) = 4.30; p = 0.0009; d = 1.15). Whether there was any adaptation transfer to the Parallel Exploration mode is unclear since the result for Parallel Testing was neither significantly different from the Serial Adaptation aftereffect (t(13) = 0.75; p = 0.47; d = 0.20) nor was it significantly different from zero (t(13) = 1.79; p = 0.097; d = 0.48). Here, it has to be noted that the results for Parallel Testing were also much more variable across participants than for the other test conditions.

### Adaptation to hand posture

Hand posture is the cue that provides slant information when using parallel exploration. The question is whether haptic adaptation is feature based or posture based. That is, are haptic object features needed in order to adapt slant perception, or does slant adaptation occur through the used hand posture even when that hand posture is not informative about object shape? The latter could have been the case during serial adaptation. For serial exploration the middle finger did not receive force feedback. This means that hand posture was unconstrained and freely chosen by participants, and did not provide information about the actual surface felt. If hand posture does indeed adapt even during serial exploration, the serial adaptation transfer effects would depend on individual participants’ randomly chosen hand postures. The hand postures thus would be a source of noise and lead to variable results for the transfer effects.

To investigate this possibility, we obtained the average posture angle α (Fig. 3A) during serial adaptation for each individual participant and correlated this with their individual after and transfer effects for the three test conditions (Fig. 3B). The average posture angle α for each participant was calculated during both the main as well as top-up serial adaptation phases. Each grey dot represents performance of an individual participant. The black lines indicate the linear regressions through these points. The offsets are informative about the average Serial Adaptation aftereffect and its transfer. The slopes indicate the influence of the hand posture as measured by the posture angle α. If the posture angle α did not have an influence on the participants’ results, the grey dots should all be horizontally aligned (regression slope of 0). This is the case when testing the aftereffect for Serial Exploration (Fig. 3B, right). A slope significantly different from zero indicates adaptation to the posture angle α. This is the case when testing for transfer effects to Parallel and Combined Exploration (e.g. Fig. 3B, left and middle). The positive slope is consistent with an “aftereffect”, not of the previous serial exploration information, but of adapting to the relative posture of the fingers. Thus, the average hand posture angle α during the Serial Adaptation phases had a major impact on the measured transfer effects. Since hand posture varied substantially across participants during Serial Adaptation (scatter of grey dots along the x-axis), this also explains the large variability in transfer effects to Parallel Testing (Fig. 2B).

Interestingly, the transfer of hand posture to Parallel and Combined Testing happens despite the fact that haptic force-feedback was provided to only one finger during Serial Adaptation. That is, during adaptation hand posture did not provide a relevant cue to surface slant, yet it still adapted. However, the aftereffect of posture was found only when posture did provide a cue to surface slant during the Parallel and Combined Testing phases. This suggests that hand posture adapts regardless of object features, but is used for estimating object features only when force-feedback from touching the object indicates it is also a relevant cue.

**Figure 4:**
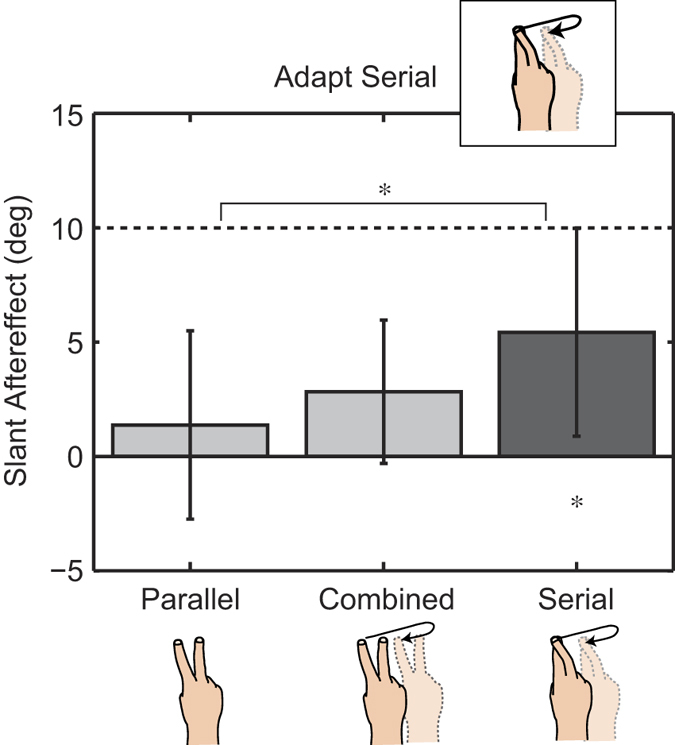
Fixed Fingers Experiment Results. Bars and error bars indicate the means and standard deviations across participants (N = 10), respectively. Participants adapted to a surface slant of 10 deg. (dashed line) using the Fixed Fingers Serial Exploration mode. The dark grey bar indicates the direct aftereffect for this condition, light grey bars the transfer conditions. The star underneath the dark grey bar indicates a significant adaptation aftereffect (comparison against zero) for the adapted Serial Exploration mode after Bonferroni-correction. The star at the top indicates a significant difference between the Serial and Parallel Testing conditions. The results show that no transfer occurs between exploration modes.

To verify the role of hand posture, we conducted a second (Fixed Fingers) Experiment. In this experiment we removed the variability in hand posture during serial exploration by instructing participants to place the middle finger on top of the index finger during both the Serial Adaptation and Serial Testing phases. Adherence to this task was verified offline using the PHANToM tracking data. The results of 2 out of a total 12 participants were removed, because their average finger distance across Serial Adaptation intervals was either too large (mean too high) or too variable (standard deviation too high) to be consistent with the instructions (the criterion for removal was 2 standard deviations away from the mean across participants). Additionally, the space in which participants could move their fingers along the sagittal plane (to and from the body) was restricted to 2.6 cm compared to 20 cm in the main experiment to further reduce variability across trials. Otherwise, the Fixed Fingers Experiment followed the procedure of the Serial Adaptation session. The results of the Fixed Fingers Experiment are shown in Fig. 4. Indeed, the inter-participant variability for Parallel Testing is reduced. Moreover, there is no transfer from Serial Adaptation to Parallel Testing (F(2, 18) = 8.7; p = 0.0023; η^2^ = 0.49; comparison Serial vs Parallel: t(9) = −3.35; p = 0.0085; d = 1.06; comparison Parallel against zero: t(9) = 1.05; p = 0.32; d = 0.33), providing further evidence for the independence of these exploration modes.

### Fast and simultaneous Serial and Parallel adaptation

Does the variation in hand posture during Serial Adaptation only explain variability across participants, or can it also account for variation in the individual trial-by-trial responses of a given participant when the Parallel Exploration mode was used? To investigate this, we analysed whether the trial-by-trial responses of the main experiment were influenced by the posture angle α adopted during the preceding top-up Serial Adaptation phase. A second factor in this analysis was the actual slant of the surface on each trial. We computed 2D Lines-of-Subjective Equality (LSE) for each participant and test-condition (using glmfit in Matlab), thus providing regression lines for individual trial responses rather than across participants. This analysis was performed for each participant individually. The obtained offsets and slopes were then averaged across participants to obtain their mean and 95% confidence intervals (Fig. 3C).

Each dot in Fig. 3C represents the actual surface slant on a given trial versus the posture angle α that was adopted during the preceding Serial Adaptation phase. The different colours represent different responses (red: left side higher, blue: right side higher). The black line represents the average LSE that separates these two response categories. For Parallel Testing the confidence interval (CI) associated with the slope of the LSE does not include zero. This indicates that the posture angle α has a significant influence on the response even on a trial-by-trial basis (Fig. 3C, left). For the Serial Testing Condition, the influence of the posture angle α is insignificant (Fig. 3C, right, CI includes zero). For Combined Testing both the aftereffects from Serial Adaptation (offset) and the posture angle α during the preceding adaptation intervals (slope) are significant (Fig. 3C, middle). This indicates that perception when using Combined Exploration is influenced by both parallel and serial cues to slant.

Importantly, the result of this analysis indicates that two forms of adaptation occur *independently, fast,* and *at the same time*. That is, during the Serial Adaptation phases, participants adapt both to the presented surface slant using serial exploration, and to the hand posture that is not linked to the actual haptically presented surface. The aftereffect from Serial Adaptation is evident only when the exploration mode during testing has a serial (movement) component (Serial and Combined Testing). The aftereffect of hand posture is evident only when it provides essential information about surface slant in the test-phase (Parallel and Combined Testing). Moreover, adaptation to hand posture occurs even on a trial-by-trial basis and thus is very fast (Fig. 3C). Together, these results provide clear evidence that serial exploration and parallel exploration are processed separately by the sensory system.

## Discussion

Here we investigated how haptic perception depends on exploration mode (parallel or serial) and if these exploration modes share a common neural pathway. If exploration modes share common neural populations, the aftereffect of one exploration mode should transfer to the other exploration mode (e.g. ref. 10). Instead, we found no transfer of adaptation across exploration modes, suggesting that serial and parallel exploration are processed by largely independent neural populations. At the very least the segregation of the neural pathways for parallel and serial exploration crosses the processing stages at which adaptation occurs, i.e. up to at least the primary somatosensory cortex^11^.

That haptic feature perception can adapt has been shown previously for haptic object size or volume (e.g. refs 15, 16, 17, 18, 19), curvature^13^,^20^,^21^,^22^,^23^,^24^,^25^, motion perception^7^,^26^,^27^,^28^,^29^ and even facial expressions^30^. Only Vogels *et al*.^22^ investigated and found transfer between static (parallel) and dynamic exploration of haptic curvature. However, in that study the whole hand was used in both the static and dynamic conditions, and thus parallel information was available in both cases and could have been responsible for the transfer effect. This is similar to our Combined condition for which we also found at least some adaptation transfer.

From a world-centred and object constancy perspective the dependence on exploration mode for determining global object shape does not seem to be a sensible strategy for perception. Naively one might have expected adaptation to occur at the level of the perceptual feature and not at the peripheral receptor level that differs between the exploration modes. We often use parallel and serial exploration simultaneously. Therefore, it would make sense to combine serial and parallel information into a unified percept early in the sensory cortex at levels that adapt to either input. This would also be in line with previous reports of transfer of adaptation across the senses^7^ and across the hands^13^,^25^. Furthermore, the way in which static (parallel) information and dynamic (serial) touch measure and weigh shape cues is highly similar^3^. A high correlation in the weighing of texture and shape cues for discriminating objects was also shown for such different exploratory procedures as contour following (serial exploration) and gripping (parallel exploration)^2^. Such similar weighting across exploration modes is useful for building stable high-level viewpoint-independent shape representations across the senses, as have been shown for vision and touch^31^,^32^,^33^,^34^. If shape perception depends too much on the exploration mode used, such high-level shape representations could hardly be consistent.

However, a common high-level perceptual representation does not necessarily mean that the information from different sources also share common low-level sensory neural populations. For instance, it has been shown that whether or not simultaneous information from vision and touch is combined into a unified percept does depend on the exploration modes used^35^. Furthermore, the current study shows that the perceptual representation of our environment, and in particular local shape information, can be highly unstable when different exploration modes are used in an intermixed fashion (Fig. 3C). Moreover, the results indicate that hand posture, an important parallel cue when touching shapes, and serial information can adapt simultaneously and independently. Interestingly, this means that parallel and serial exploration can potentially provide incongruent information even when the sensory information is extracted concurrently from the exact same object. A surprising notion given that we often move the whole hand across surfaces to explore them, and we generally seem unaware of such inconsistencies. This suggests that the Central Nervous System somehow discards such perceptual incongruences, for instance by integrating the independent estimates from the separate exploration modes. If two sources of information are independent in terms of the noise sources, integrating them using a reliability based weighting scheme (see e.g. ref. 5) would lead to more reliable estimates. Thus a certain level of independent processing and thus processing noise can be beneficial for perception. Future research in which the exploration movements are more accurately controlled to avoid noise from movement variations should address if the exploration modes are indeed integrated using such a reliability-based scheme.

The distinction between exploration modes for haptic perception appears to fit in well with a more body-centred perspective. For haptic exploration, sensing the position and movement of our limbs (proprioception) plays a crucial role for combining touch sensations from different spatial locations into one coherent percept. For proprioception it has long been debated whether position and movement sensing are processed by the same or separate mechanisms (see e.g. refs 36, 37, 38 for reviews). However, the evidence for separate mechanisms seems to be based mostly on peripheral mechanoreceptor responses in the skin and muscles. How these different sources of information are combined by the Central Nervous System is less clear. Recent studies indicate that the information from different mechanoreceptors is combined very early in the somatosensory cortex^4^,^39^,^40^. This would suggest that sensory information from different exploration modes, which each will activate a combination of mechanoreceptors^40^, might be treated by the same neural population, at least at some level in the cortex. However, the current results provide very strong evidence for separate processing of hand posture (parallel exploration) and hand movement (serial exploration) throughout the neural processing pipeline for haptic perception, since they can independently adapt at the same time and no transfer was found to the other exploration mode. This also fits with previous studies that suggest a similar segregation for motor control^41^,^42^,^43^,^44^,^45^ and the idea of multiple sub-modality pathways^4^. Note however, that the segregation between serial and parallel haptic information cannot simply be due to a difference in active versus passive touch. Serial information has been shown to transfer between passive (object moving) and active (hand moving) conditions^13^, and the active component only seems to lead to increased discrimination thresholds^46^. If passive and active haptic perception do not adapt independently, why does this seem to be the case for the different modes of serial and parallel exploration? A potential explanation might be that positioning the hand for haptic exploration depends on the control and sensation across multiple joints and muscles. If the system receives a recalibration signal, how does it know which sub-system to adjust (i.e. which joint etc. is at fault)? For passive and active serial exploration, the calibration signal can still be attributed to the same cause if the stimuli are similar enough. However, hand posture and movement rely on different combinations of muscle and joint signals and the error may thus be attributed to independent causes. This fits with hand angle production biases differing if the hand posture is produced by wrist movements or elbow movements only^47^. To allow correcting for different causes of misalignment with respect to the world, independent mode-specific adaptation can be beneficial.

Interestingly, the current study shows that the segregation between posture (parallel exploration) and movement (serial exploration) likely crosses the majority, if not all, of the sensory neural subsystems necessary for haptic perception. If information from serial and parallel exploration is combined, it seems this occurs only at down-stream processing levels at which no adaptation occurs. This means that early sensory populations including the primary somatosensory cortex S1 likely can be ruled out (see in ref. 11). Future work should verify this by investigating the neural activation of neurons in S1 when both movement and posture information is available. Based on the present results we conclude that, rather than being merged into a unitary pipeline, haptic information is processed by separate serial and parallel sub-modality neural pathways that operate in independent fashion.

## Additional Information

**How to cite this article**: van Dam, L. C. J. *et al*. Haptic adaptation to slant: No transfer between exploration modes. *Sci. Rep.* **6**, 34412; doi: 10.1038/srep34412 (2016).
